# Rituximab-Associated Liver Toxicity Without Known Viral Reactivation

**DOI:** 10.7759/cureus.64331

**Published:** 2024-07-11

**Authors:** Taha Huda, Fawaz Hussain, Hariharasudan Mani, Shereen M Gheith, Savitri Skandan

**Affiliations:** 1 Internal Medicine, HCA Florida Bayonet Point Hospital, Hudson, USA; 2 Internal Medicine, University of South Florida Morsani College of Medicine, Tampa, USA; 3 Hematology and Medical Oncology, Lehigh Valley Health Network, Allentown, USA; 4 Hematopathology and Clinical Laboratory Medicine, Lehigh Valley Health Network, Allentown, USA

**Keywords:** hepatotoxicity, transaminitis, marginal zone b cell lymphoma, drug-induced liver injury (dili), rituximab

## Abstract

Rituximab is a targeted immunotherapeutic agent that has demonstrated efficacy in treating CD20+ B-cell neoplasms as well as other lymphoproliferative and autoimmune disorders. A major adverse effect of rituximab is hepatocellular injury attributed to hepatitis B viral reactivation, necessitating viral titers before treatment. In this case report, we illustrate the rare presentation of a patient with marginal zone B-cell lymphoma who experienced symptomatic liver injury with a peak 15-fold aminotransferase elevation following his first dose of rituximab, without evidence of viral reactivation.

## Introduction

Rituximab is a chimeric mouse/human anti-CD20 monoclonal antibody that has been used for a variety of diseases, including lymphoproliferative disorders and autoimmune conditions [1]. Given its lengthy two-decade use for a myriad of conditions, many side effects have been elucidated; regarding hepatotoxicity, the standard side-effect profile includes a mild elevation in aminotransferases (i.e., less than five times the upper limit of normal) in 10-15% of the population. In rare cases, rituximab has been linked with viral reactivation of hepatitis or other viruses [1]. However, few case reports have been put forth detailing evidence of rituximab-associated hepatocellular damage after ruling out known viral or autoimmune causes. These cases range from asymptomatic or symptomatic manifold increases in aminotransferases, which either self-resolve [2-6] or progress to acute liver failure [7]. These rare responses necessitate further understanding of the characteristics of this drug-induced liver injury.

## Case presentation

**Figure 1:**
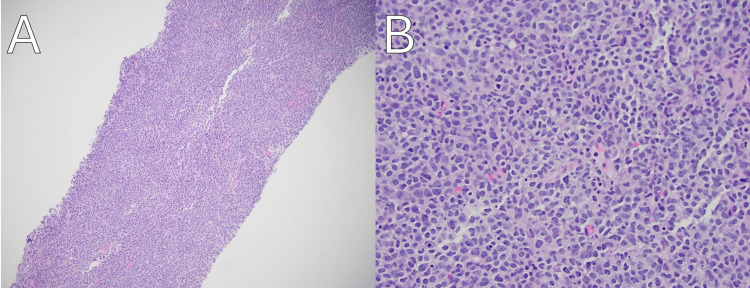
Axillary lymph node biopsy showcasing marginal zone lymphoma in this case at 10x magnification (A) and 40x magnification (B), hematoxylin-eosin stain.

**Figure 2:**
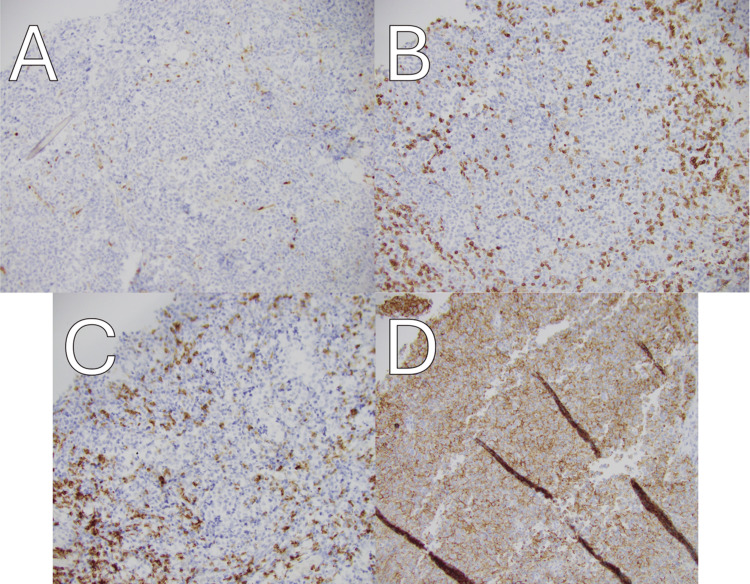
Immunohistochemistry demonstrates lymphoma cells negative for BCL-1 (A), CD3 (B), CD5 (C), and positive for CD20 (D) at 20X magnification.

A 52-year-old male patient with a past medical history of stable Sjogren’s syndrome managed intermittently with hydroxychloroquine for over ten years was referred to our hematology clinic for axillary lymphadenopathy and newly diagnosed bicytopenia with an absolute neutrophil count of 1200 × 109/L and a platelet count of 91000 × 109/L. In the prior three months, he had lost approximately 10% of his body weight and experienced worsening fatigue and drenching night sweats. CT imaging showed lymph node involvement on both sides of the diaphragm and a splenomegaly of 16 cm. An axillary lymph node biopsy revealed histopathological findings consistent with marginal zone B-cell lymphoma (Figure 1). The immunohistochemistry of the biopsy was negative for BCL1, CD3, CD5, and CD10 and positive for CD20 (Figure 2). The BCL-6 stain highlighted residual irregular and disrupted germinal centers that were BCL-2 negative, with associated CD21 follicular dendritic cells and a prominent Ki-67. CD138 stain highlighted frequent plasma cells that were kappa light chain restricted. The lambda light chain was also largely negative. MYD88 was negative per polymerase chain reaction (PCR). His baseline liver function tests recorded two months prior to treatment included an aspartate transaminase (AST) of 35 U/L, an alanine transaminase (ALT) of 58 U/L, an alkaline phosphatase (ALP) of 81 U/L, and a total bilirubin (TBili) of 0.7 mg/dL. A week before treatment, hepatitis B and C serology were negative. Immunoglobulin levels were within normal limits, and a bone marrow biopsy was negative for lymphoma involvement. The patient was thus started on single-agent rituximab treatment, dosed at 375 mg/m2. During the infusion, the patient experienced nausea, vomiting, and a low-grade fever, which improved following antiemetics and acetaminophen; he was otherwise asymptomatic.

**Figure 3:**
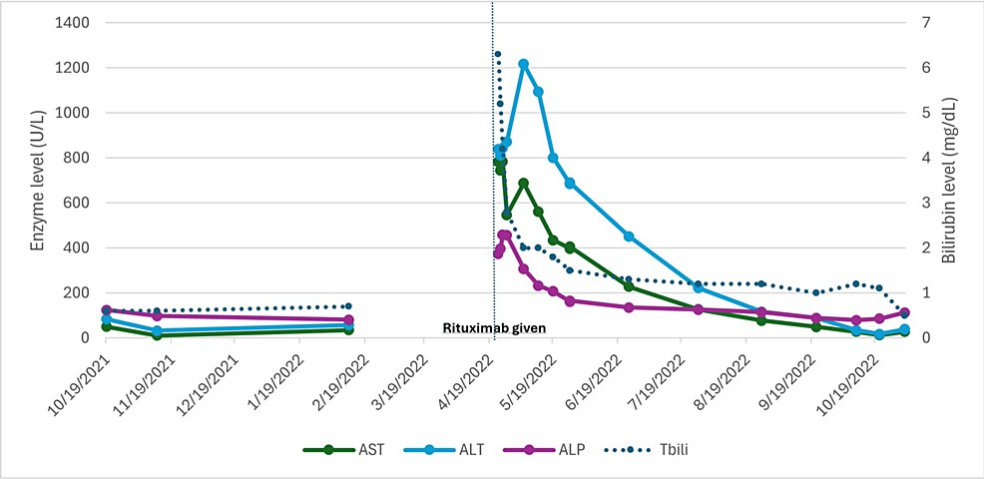
Graph showcasing peak of acute liver injury following rituximab administration on 4/22/2022. The left vertical axis represents the units for the enzymes (AST, ALT, and ALP), whereas the right vertical axis represents the units for total bilirubin. AST: aspartate transaminase; ALT: alanine transaminase; ALP: alkaline phosphatase; Tbili: total bilirubin

The following day, the patient presented to the emergency department for evaluation of mild epigastric pain, jaundice, and dark brown urine. The patient denied prior alcohol use, and his blood alcohol level on admission was undetectable. He had no history of recent travel. The patient was not on medications other than acetaminophen and hydroxychloroquine and denied starting any medications within the prior three months. His symptoms from Sjogren's disease, namely, sicca, remained unchanged. His physical examination was remarkable for scleral icterus and generalized jaundice, but his vital signs and abdominal exam were otherwise unremarkable. The oncology and gastroenterology teams were consulted, and the patient was admitted to the hospital. He was found to have an AST of 782 U/L, ALT of 837 U/L, ALP of 372 U/L, total bilirubin of 6.3 mg/dL, direct bilirubin of 5.4 mg/dl, and lactate dehydrogenase of 1244 U/L. These liver function tests were all within normal limits when they were last checked (Table 1, Figure 3). The R factor for liver injury in this case was calculated at 6.8, suggesting hepatocellular injury. Acetaminophen levels were within normal limits. A CT abdomen/pelvis with contrast was performed, which was unremarkable, and hepatobiliary ultrasound showed no evidence of cholelithiasis or biliary duct dilatation. The patient’s viral serologies prior to the rituximab infusion and repeat serologies during his admission were all negative for antibodies to hepatitis A, B, and C, cytomegalovirus (CMV), and Epstein-Barr virus (EBV). Furthermore, antimitochondrial, anti-smooth muscle and anti-liver kidney antibodies were negative. His anti-Ro and anti-La levels six months prior to this event were 118 IU and 27 IU, respectively, and were found to be at 115 IU and <20 IU, respectively, when he presented. Given his liver injury, all potentially hepatotoxic medications, including acetaminophen and hydroxychloroquine, were discontinued. 

**Table 1:** Liver function tests before and after rituximab infusion. AST: aspartate transaminase; ALT: alanine transaminase; ALP: alkaline phosphatase

Liver function tests	Baseline from 2 months prior	One day following rituximab infusion	Normal lab values
AST	35 U/L	782 U/L	15-37 U/L
ALT	58 U/L	837 U/L	12-78 U/L
ALP	81 U/L	372 U/L	45-117 U/L
Total bilirubin	0.7 mg/dl	6.3 mg/dl	0.20-1.00 mg/dl
Lipase	N/A	206 U/L	7-60 U/L

Over the next 48 hours, the patient was given intravenous fluids and kept under observation. His total bilirubin trended downward, but his AST and ALT remained elevated 15 times the normal limit. Despite this acute elevation, the patient's presenting symptoms had resolved. Therefore, he was discharged home and followed up in the outpatient clinic. The patient did not receive any further rituximab infusions, and his liver enzymes gradually trended downward until returning within normal limits three months later. His Revised Electronic Causality Assessment Method (RECAM) score, a computerized causality assessment tool for drug-induced liver injury (DILI) diagnosis [8], was 9, indicating a high probability of causality. Given the proximity to the rituximab infusion, that his presenting symptoms had self-resolved, and that the above work-up did not point to another etiology, the patient was diagnosed with drug-induced liver injury related to rituximab and discharged home. The patient had worsening lymphadenopathy on his four-month follow-up CT imaging, along with worsening B symptoms. Thus, he was transitioned to a different treatment regimen with cyclophosphamide, vincristine, and prednisone (CVP). He did not develop further liver injury while on CVP; however, there was a poor treatment response on this regimen with the progression of his lymphadenopathy. The re-biopsy of lymph nodes was consistent with marginal zone lymphoma without evidence of transformation. He was transitioned to zanubrutinib (320 mg daily) with good control of his disease.

## Discussion

Given the acute development of symptoms following this patient's rituximab infusion, the absence of other new hepatotoxic agents, and negative viral and autoimmune serologies, this case is most consistent with rituximab drug-induced liver injury (DILI). This diagnosis is also reinforced by the improvement of the patient’s liver function tests and the lack of recurrence after discontinuing rituximab. Our patient’s presentation is distinct from the standard cases of liver injury following rituximab infusion, which typically show mild elevations in transaminases or evidence of viral reactivation, most commonly involving hepatitis B. One important limitation of this case report is the lack of reported liver enzyme data prior to the infusion, though it is again crucial to note the close temporal-acute association of his clinical symptoms with the infusion in the absence of other causes.

On comparing this patient to the other cases of rituximab hepatotoxicity without known viral reactivation noted in Table 2 [2-7], we noted that our patient uniquely had a much longer time to hepatotoxicity resolution of three months, unlike most cases, which resolved within two weeks. This three-month resolution period was shared with one other case who also had Sjogren’s syndrome [5], raising further concern about an exacerbation of DILI in patients with autoimmune disorders. However, before and after our patient’s episode of hepatotoxicity, the levels of his anti-Ro and anti-La antibodies were unchanged. Another difference noted in this case presentation was the occurrence of DILI immediately following the first induction of rituximab, which had typically occurred on second or third infusions in most other reported cases. Note that no rechallenge of the rituximab was performed in any of the cases following the suspicion of DILI to avoid further liver toxicity.

**Table 2:** Cases reporting rituximab-induced liver toxicity without known viral reactivation. AST: aspartate transaminase; ALT: alanine transaminase; TBili: total bilirubin

Author, Year	Age, Sex	Disease treated with rituximab	Time to onset of reported rituximab-induced liver toxicity	Symptoms	Peak liver function tests	Time to liver function test return to baseline
Toprak, 2012 [2]	50, F	Rai Stage II chronic lymphocytic leukemia	1 day after 3^rd^ dose	Asymptomatic	AST: 611 U/L, ALT: 507 U/L, TBili: 0.3 mg/dl	6 days
Del Prete, 2010 [3]	38, M	Immune thrombocytopenic purpura	3 days after 3^rd^ dose	Low-grade fever, aches, jaundice	AST: 104 U/L, ALT: 256 U/L, TBili: 12 mg/dl	7 days
Upadhyay H, 2013 [4]	52, M	Stage IV follicular lymphoma	1 week after 1^st^ dose	Scleral icterus	AST: 2089 U/L, ALT: 2165 U/L, TBili: 10.5 mg/dl	2 weeks
Galiatsatos, 2010 [5]	40, F	Mucosa-associated lymphoid tissue lymphoma	Following 2^nd^ dose	Asymptomatic	AST: 855 U/L, ALT: 1,151 U/L TBili: “normal’	3 months
Joy, 2019 [6]	66, F	Pemphigus foliaceus	1 week after 1^st^ dose	Asymptomatic	AST: 106 U/L, ALT: 310 U/L, TBili: “normal”	1 week
Qazilbash, 2005 [7]	21, F	Autoimmune hemolytic anemia following bone marrow transplantation for chronic myeloid leukemia	3 days after 1^st^ dose	Acute liver failure	AST: >2000 U/L, ALT: >2000 U/L, TBili: 62 mg/dl	N/A

Identifying the mechanism for this reaction is elusive, especially given that a liver biopsy was not performed due to the patient’s symptomatic improvement and spontaneous improvement in his transaminases. As a liver biopsy is primarily indicated in the case of overlap syndromes, only two of the reported rituximab-attributed liver toxicity cases in Table 2 included a liver biopsy. In Galiatsatos et al. [5], an asymptomatic case was found to have lobular infiltration by lymphocytes consistent with acute autoimmune hepatitis, whereas in Qazilbash et al. [7], the case of acute liver failure was found to have hepatocellular necrosis in the periportal zone. The lack of multiple examples of histopathology unfortunately limits understanding of the mechanism behind this interaction. Nevertheless, when approaching DILI, mechanisms are grouped as direct hepatotoxicity or indirect hepatotoxicity. In this case, direct hepatotoxicity would be extremely unlikely, given that antibodies do not generate toxic intermediates. However, indirect hepatotoxicity is feasible, where a processed peptide may have acted as a foreign epitope and led to an immune response. Consistent with this mechanism, the serum of one case who experienced cholestatic injury following rituximab infusion was found to have cross-reacting antibodies to Fab2, indicating a potential subclinical autoimmune mechanism for these findings [9].

## Conclusions

In conclusion, this case presentation contributes to the growing understanding of rituximab-induced liver injury in the absence of viral reactivation and highlights this very rare risk of utilizing rituximab. It also emphasizes the importance of monitoring liver function tests in patients receiving rituximab and educating them on relevant symptoms that may warrant further medical evaluation. Further studies are still needed to explore the potential mechanisms of hepatocellular damage from rituximab. 

## References

[1] (2024). Rituximab. https://www.ncbi.nlm.nih.gov/books/NBK548249/.

[2] Toprak SK, Karakuş S (2012). Rituximab-related reversible hepatocellular damage. Turk J Haematol.

[3] Del Prete CJ, Cohen NS (2010). A case of rituximab-induced hepatitis. Cancer Biother Radiopharm.

[4] Upadhyay H, Sherani K, Vakil A, Cervellione K, Patel A (2013). Acute hepatocellular damage secondary to rituximab-induced hepatitis. Am J Gastroenterol.

[5] Galiatsatos P, Assouline S, Gologan A, Hilzenrat N (2020). Rituximab-induced autoimmune hepatitis: a case study and literature review. Can Liver J.

[6] Joy N, Sobhanakumari K, Celine MI, Mathew R, Athira S (2019). Rituximab induced transaminitis in pemphigus foliaceus. J Skin Sex Transm Dis.

[7] Qazilbash MH, Qu Z, Hosing C, Couriel D, Donato M, Giralt S, Champlin R (2005). Rituximab-induced acute liver failure after an allogeneic transplantation for chronic myeloid leukemia. Am J Hematol.

[8] Hayashi PH, Lucena MI, Fontana RJ (2022). RECAM: A new and improved, computerized causality assessment tool for DILI diagnosis. Am J Gastroenterol.

[9] Latus J, Klein R, Koetter I (2013). Cholestatic liver disease after rituximab and adalimumab and the possible role of cross-reacting antibodies to Fab 2 fragments. PLoS One.

